# Learning Dendritic-Neuron-Based Motion Detection for RGB Images: A Biomimetic Approach

**DOI:** 10.3390/biomimetics10010011

**Published:** 2024-12-28

**Authors:** Tianqi Chen, Yuki Todo, Zhiyu Qiu, Yuxiao Hua, Delai Qiu, Xugang Wang, Zheng Tang

**Affiliations:** 1 Division of Electrical Engineering and Computer Science, Kanazawa University, Kakuma-machi, Kanazawa 920-1192, Ishikawa, Japan; chentianqi@stu.kanazawa-u.ac.jp (T.C.); qiuzy1916@stu.kanazawa-u.ac.jp (Z.Q.); sh13818971028@gmail.com (Y.H.); 2 Faculty of Electrical, Information and Communication Engineering, Kanazawa University, Kakuma-machi, Kanazawa 920-1192, Ishikawa, Japan; 3 Brain Science Institute, Jilin Street, Jilin 132013, China; dlqiu@jimu.edu.cn; 4 Beijing Euler Cognitive Intelligence Technology Co., Ltd., Beijing 100097, China; wangxugang@gve.ai; 5 Institute of AI for Industries, Chinese Academy of Sciences Nanjing, 168, Tianquan Road, Nanjing 211135, China; 6 School of Computer Engineering and Science, Shanghai University, Shanghai 200444, China

**Keywords:** artificial visual system, neural network, dendritic neuron, motion direction detection, deep learning

## Abstract

In this study, we designed a biomimetic artificial visual system (AVS) inspired by biological visual system that can process RGB images. Our approach begins by mimicking the photoreceptor cone cells to simulate the initial input processing followed by a learnable dendritic neuron model to replicate ganglion cells that integrate outputs from bipolar and horizontal cell simulations. To handle multi-channel integration, we utilize a nonlearnable dendritic neuron model to simulate the lateral geniculate nucleus (LGN), which consolidates outputs across color channels, an essential function in biological multi-channel processing. Cross-validation experiments show that AVS demonstrates strong generalization across varied object–background configurations, achieving accuracy where traditional models like EfN-B0, ResNet50, and ConvNeXt typically fall short. Additionally, our results across different training-to-testing data ratios reveal that AVS maintains over 96% test accuracy even with limited training data, underscoring its robustness in low-data scenarios. This demonstrates the practical advantage of the AVS model in applications where large-scale annotated datasets are unavailable or expensive to curate. This AVS model not only advances biologically inspired multi-channel processing but also provides a practical framework for efficient, integrated visual processing in computational models.

## 1. Introduction

The visual system is the primary source of information for mammals, contributing approximately 80% of the sensory data received from the external world [1]. At the forefront of this processing is the retina, which handles a significant portion of visual information by filtering and converting light into neural signals [2]. However, the more complex and essential stages of visual processing occur beyond the retina, particularly in the lateral geniculate nucleus (LGN) and the primary visual cortex (V1). These brain regions play a critical role in classifying and interpreting visual data, which in turn influence behaviors and decision-making [3].

One of the key functions of the visual system is the detection of motion direction, which is crucial for responding to dynamic environments. Previous research, including our own, has demonstrated that motion direction detection is a learnable process [4]. In this context, bio-inspired models have emerged as efficient alternatives, capable of addressing the limitations of traditional computational approaches. Bio-inspired models often achieve faster and more accurate image analysis than traditional computational approaches when simulating this functionality [5]. Such models leverage biological principles to enhance biological reasonability and efficiency, distinguishing themselves from purely data-driven methods. This is because these models can mimic the efficiency and adaptability of biological systems. In particular, our bio-inspired model is based on the dendritic neuron model, a biologically plausible structure that mimics the tree-like processing units found in the brain [6]. Dendritic computation allows for the integration of multiple synaptic inputs across different spatial locations, a feature particularly important in tasks such as motion detection and orientation selectivity [7]. Dendrites can integrate excitatory and inhibitory inputs at various locations on the dendritic tree, allowing for nonlinear signal processing that enhances the model’s capacity for spatiotemporal information processing [8]. This structure enables our model to simulate how visual information is processed in the brain, offering higher biological realism and computational efficiency. In addition to motion detection, the visual system’s ability to process information across multiple channels—such as color and intensity—plays a pivotal role in human perception. The LGN and V1 are responsible for classifying visual input into different optic channels, such as the red, green, and blue (RGB) channels in humans [9]. Studies have further established that color information is just as crucial as motion or shape information for understanding the environment [10].

Despite the success of our previous model in handling single-channel information (such as grayscale images), natural visual scenes involve multi-channel inputs. Specifically, cone cells in the retina detect different wavelengths of light corresponding to RGB channels [1]. These signals, processed by bipolar and horizontal cells in the retina, are then classified by the LGN before reaching higher cortical areas for interpretation [11]. In our work, we have simplified this biological process into an RGB model for computational simulation. Our previous model, based on the dendritic neuron model, effectively processed motion direction detection in single-channel grayscale images [12]. This multi-layered biological processing serves as the foundation for our model’s design, highlighting its alignment with natural vision pathways. Dendritic models are particularly suited for handling such tasks due to their ability to integrate spatially distributed inputs, making them more efficient for spatiotemporal processing. However, handling multi-channel visual information, such as RGB inputs for color processing, required an updated version of the model with a more complex structure and additional functions.

While convolutional neural networks (CNNs) are widely regarded as powerful tools for classification and detection tasks, including motion direction detection, they have significant limitations [13,14]. CNNs require vast amounts of training data and computational resources, making them inefficient for certain applications [15]. Furthermore, their lack of interpretability limits their application on biologically relevant insights required situations. In contrast, bio-inspired models offer a more resource-efficient approach. These models, especially the dendritic neuron model, through their biological realism and computational advantages, achieve high accuracy with reduced training data and computational costs [14]. The dendritic neuron model, with its biologically grounded structure, achieves robust performance with fewer data and training time. This allows the model to handle complex visual tasks while maintaining computational efficiency, supporting its overall design.

To address the limitations of single-channel bio-inspired models, we propose an enhanced model. This novel model integrates the functionality of cone cells and LGN’s multi-channel processing with our previous work on motion direction detection. The dendritic structure’s capacity for nonlinear integration of signals makes this model ideal for handling the complexity of RGB image processing, providing a biologically plausible yet efficient solution. Additionally, we analyze the model’s performance across diverse datasets to understand its real-world applicability and its ability to generalize across different visual scenarios. This new model effectively handles three-channel RGB images with improved accuracy and biological plausibility. Additionally, the model offers strong robustness and reduced computational costs, making it a promising solution for a variety of computer vision tasks.

## 2. Method

### 2.1. Related Works

Dendritic computation plays a critical role in biological and bio-inspired models for visual processing. Dendrites facilitate the integration of excitatory and inhibitory inputs across spatial domains, enabling nonlinear signal processing capabilities [16]. This capacity is particularly essential in motion direction detection and directional selectivity, as it allows neurons to process spatiotemporal patterns effectively [7]. Models inspired by these mechanisms highlight the efficiency of localized nonlinear computation, which is crucial for recognizing patterns amid variability in orientation and motion direction. Recent advances in dendritic models have applied these biological principles to computational frameworks. It was demonstrated that incorporating dendritic structures enhances spatial and temporal feature extraction, reducing computational overhead while improving model precision [17]. Such designs emphasize localized nonlinear processing to mimic fine-grained biological feature recognition, making them suitable for tasks requiring motion direction detection.

Furthermore, dendritic-inspired neuron models allow efficient information integration, providing robust solutions for processing high-dimensional visual data [8]. A key distinction arises in how different dendritic-inspired models handle visual inputs. For example, the On–Off model of dendritic computation is employed in a research study that integrates excitatory and inhibitory pathways to replicate the selective response mechanisms seen in biological neurons [18]. This structure is particularly adept at detecting contrasting motion signals by balancing these inputs. On the other hand, we also designed models that operate without LGN preprocessing, directly handling grayscale inputs, which simplifies the computational pipeline at the cost of some biological plausibility [19]. These differences highlight the trade-offs between biological alignment and computational efficiency.

By leveraging the unique computational properties of dendrites, our work extends the principles of motion direction detection to multi-channel RGB image processing. This involves mimicking the localized computation of ganglion cells, which integrate signals through their dendritic arbor to detect motion direction effectively [20]. Our proposed model, inspired by these principles, addresses the challenges of multi-channel data processing while maintaining computational efficiency and scalability.

### 2.2. RGB Image Processing

Image processing is a critical function in the field of computer vision, enabling systems to analyze and interpret visual data. In digital imaging, most images are represented as 3-channel images, with each channel corresponding to different color information. These channels can be represented in various color models, such as RGB (Red, Green, Blue), VHS (Hue, Saturation, Value), and others, depending on the application and the specific needs of the processing task [21].

**Figure 1:**
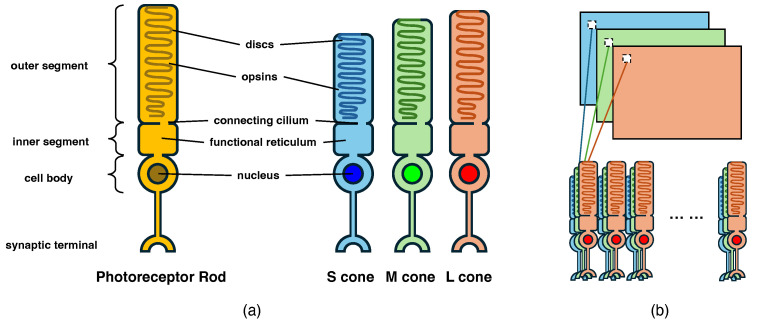
The structure of rod and cone photoreceptor cells is shown in (**a**), while (**b**) illustrates how a single pixel in a 3-channel image is processed by the cone cells, with each cone type responding to a different wavelength corresponding to the three color channels.

In biological systems, the processing of visual information is closely tied to the wavelengths of the light entering the eye [22]. This biological mechanism is conceptually similar to the RGB model used in digital image processing. The photoreceptor cells in the retina, particularly the cone cells, are responsible for detecting light stimuli across different wavelengths. Each type of cone cell is sensitive to a specific range of wavelengths, roughly corresponding to the red, green, and blue channels in digital imaging [23]. Once the photoreceptors detect light, the visual information undergoes further processing within the retina [24]. The bipolar cells and horizontal cells are the primary intermediaries, refining and transmitting the information to higher visual centers [25]. After this retinal processing, the signals are conveyed to the lateral geniculate nucleus (LGN), where the 3-channel information is further integrated and processed [26]. The LGN plays a pivotal role in organizing and routing this information to the primary visual cortex (V1), where more complex detection and interpretation tasks, such as object recognition and motion detection, are performed [27]. Thus, the biological pathway for processing multi-channel visual information shares structural similarities with computational models like RGB, where pixel values in digital images mirror the color-sensitive photoreceptor responses to light in biological systems. In this study, Figure 1a illustrates the structure of rods and cone cells, which are capable of processing multi-channel input information. Since rods primarily handle the detection of light intensity, we focus on using the three types of cone cells (S, M, L cones), each sensitive to different wavelengths, to simulate the RGB model’s three channels. Figure 1b demonstrates how S, M, and L cone cells scan the intensity of each pixel and assign the information to the respective cone cells for processing, effectively decomposing and integrating color information. This distributed processing approach parallels the allocation of color channels in digital image processing, allowing both biological and computational systems to integrate and interpret color and light signals to produce meaningful visual representations.

### 2.3. Vertical and Horizontal On–Off Response Based on Bipolar Cells and Horizontal Cells

The detection of the direction of an object’s motion begins when the retina receives light stimuli, including brightness, darkness, and spatiotemporal color changes, which are then converted into electrical signals by the photoreceptor cells [28,29]. In the case of RGB image processing, the model must address the added complexity of multi-channel inputs. Similar to the approach used in grayscale image processing, we extend the use of horizontal and bipolar cell-like mechanisms to handle each of the three color channels (red, green, and blue) individually.

In computational models, an A×B two-dimensional RGB image is typically represented as three separate A×B matrices, each corresponding to one of the red, green, and blue channels. Each pixel in the image contains three values, one for each channel, representing the intensity of red, green, and blue components. These values, for example, a pixel located at (a,b), which means the *a*th row and *b*th column and is denoted by $x_{\left(a,b\right)}^{R}$, $x_{\left(a,b\right)}^{G}$, and $x_{\left(a,b\right)}^{B}$ for the red, green, and blue channels, respectively, range from 0 to 255. A value of 0 represents the absence of the color (black for that channel), while 255 represents full intensity. Intermediate values represent varying intensities of the respective color. Each pixel at location (a,b) serves as the center pixel of the receptive field at that location but also functions as a surrounding pixel for other nearby coordinates. This multi-channel representation, similar to the grayscale model but extended to three dimensions, allows for the storage and processing of more complex images. The representation format, commonly known as uint8, simplifies the storage and manipulation of RGB images in digital systems, allowing efficient use of memory and processing resources.

**Figure 2:**
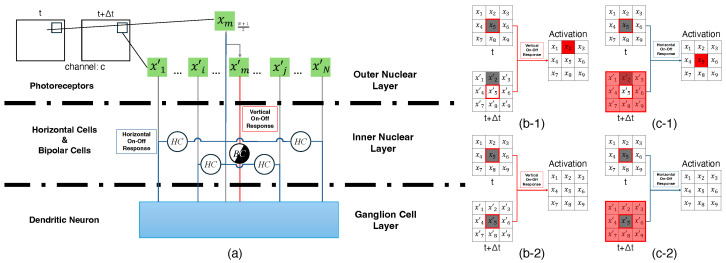
(**a**) Illustrates the three nuclear layers of the retina: the outer layer (photoreceptors), the inner layer (comprising bipolar and horizontal cells), and the ganglion cell layer, which together form the basic structure of the mammalian visual system. (**b-1**) Depicts the ‘on’ response of the vertical On–Off response of the center pixel. (**b-2**) Displays the ‘off’ response of the vertical On–Off response of the center pixel. (**c-1**) Shows the ‘on’ response of the horizontal On–Off response to motion in the ‘up’ direction. (**c-2**) Represents the ‘off’ response of the horizontal On–Off response in the absence of motion.

Each channel is processed using analogous neurons to horizontal cells (HCs) and bipolar cells (BCs), with the same vertical and horizontal On–Off response mechanisms applied across all channels. In our model, for each color channel, the photoreceptors—analogous to the cone cells in biological systems—convert light into electrical signals that represent the pixel intensities of that channel. These signals are then processed by horizontal and bipolar cells to detect changes in pixel intensity, allowing for the identification of motion and object boundaries. For instance, in Figure 2a, channel c of a 3-channel motion image sequence of size A×B (comprising an image at time *t* and a subsequent image at t+Δt) is analyzed. Here, $x_{m}$ represents the center pixel at location (a,b) in the image at time *t*, while $x_{1}^{\prime }$ to $x_{N}^{\prime }$ denote all pixels in the receptive field in the same location at t+Δt (*N* is the size of a single receptive field). Motion information is processed within the inner nuclear layer, where horizontal and bipolar cells handle horizontal and vertical On–Off responses, respectively, and the resulting signals are normalized for subsequent processing. These signals are then conveyed to the ganglion cell layer, where they are further processed by ganglion cells.

For vertical On–Off response, bipolar cells process the signal intensity in each channel to detect differences between the center pixel before motion and its value after motion. Bipolar cells output binary responses based on whether the pixel intensity exceeds a given threshold, $\epsilon_{V}$. The receptive field is a 3×3 grid, and for each color channel, the output of the bipolar cell, $B_{(a,b)h}^{c}\left(h=5\right)$, is defined as

(1) $$B_{(a,b)5}^{c}=\left\{\begin{array}{c} 1,(|x_{(a,b)5}^{c}-x_{{\left(a,b\right)}^{\prime }5}^{c}|>\epsilon_{V})\\ 0,(|x_{(a,b)5}^{c}-x_{{\left(a,b\right)}^{\prime }5}^{c}|<\epsilon_{V}) \end{array}\right.,$$

where (a,b) is the location of the center pixel of the receptive field before motion; ${\left(a,b\right)}^{\prime }$ is the location of the center pixel after motion; $x_{(a,b)5}^{c}$ represents the intensity of the center pixel at (a,b) in channel *c*; $x_{{\left(a,b\right)}^{\prime }5}^{c}$ represents the intensity of the center pixel at ${\left(a,b\right)}^{\prime }$ in channel *c*; and i=5 refers to the center pixel in the 3×3 receptive field. Figure 2b illustrates the vertical On–Off response mechanism. When motion occurs within the receptive field, as shown in Figure 2(b-1), the corresponding bipolar cell is activated. In contrast, when no motion is detected, as in Figure 2(b-2), no activation of the bipolar cells occurs.

For horizontal On–Off response, horizontal cells in our model are responsible for processing the differences between the pixel intensities in the surrounding areas compared to the center pixel in each channel. The pixel intensity differences are compared to a threshold, $\epsilon_{H}$, for horizontal cell activation. In the receptive field of a 3×3 grid, each pixel in this grid is compared to the center pixel value, which can be defined as

(2) $$H_{(a,b)h}^{c}=\left\{\begin{array}{c} 1,(|x_{(a,b)5}^{c}-x_{{\left(a,b\right)}^{\prime }i}^{c}|<\epsilon_{H})\\ 0,(|x_{(a,b)5}^{c}-x_{{\left(a,b\right)}^{\prime }i}^{c}|>\epsilon_{H}) \end{array}\right.,$$

where *i* represents the index of the pixel in the receptive field grid, where i=5 refers to the center pixel and i≠5 refers to the surrounding pixels. Figure 2c illustrates the horizontal On–Off response mechanism. When motion occurs within the receptive field in the ‘up’ direction (e.g., from the center pixel $x_{5}$ to the pixel $x_{2}^{\prime }$ above it), as shown in Figure 2(c-1), the corresponding horizontal cell is activated. Conversely, when no motion is detected, as in Figure 2(c-2), no activation of the horizontal cells takes place.

By processing each RGB channel independently using the same horizontal and vertical On–Off response mechanisms, the model is able to effectively identify motion and color boundaries in multi-channel images.

### 2.4. Learning Dendritic Neuron Model to Ganglion Cells

Ganglion cells play a pivotal role in the visual processing system, serving as the final stage in the retinal network before transmitting information to the brain via the optic nerve. They receive synaptic inputs from BCs and HCs. This combination allows ganglion cells to detect and encode various visual features such as orientation, motion direction, and contrast. In particular, the dendrites of ganglion cells are crucial in the integration of these inputs, enabling complex spatial and temporal processing. The structure and arrangement of the dendrites allow ganglion cells to selectively respond to specific visual stimuli, making them essential for feature detection in the visual system.

**Figure 3:**
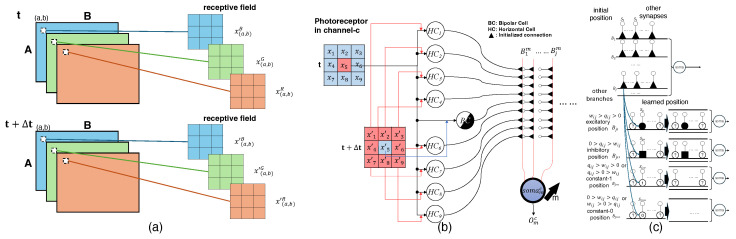
(**a**) Represents the scanning positions from each channel following the processing described in Figure 2a. Each separated channel, corresponding to the cone cells, has its own receptive field of size 3×3. (**b**) Illustrates how the soma processes the signals, where $soma_{m}^{c}$ corresponds to direction $O_{m}^{c}$ in channel c. (**c**) The impact of weights and biases within the synapses on the connection states between the synapse and the dendritic branches is explored. These states are classified into four categories: excitatory, inhibitory, constant-1, and constant-0. Due to the nonlinear algorithm employed by the dendritic model, these synaptic states influence the connections from the branches to the soma. The relationship between the various states, and the weights and biases relative to zero, is depicted in the accompanying figure.

The dendritic neuron model leverages dendritic functionality crucial for computer vision tasks since it has strong biological alignment [30]. Since the dendrites play a crucial role in motion detection, the employment of dendritic neuron models to ganglion cells aims to be a reasonable design [20]. As a result, we aim to replicate the functionality of ganglion cells by utilizing a dendritic neuron model, with a particular focus on detecting the orientation of visual stimuli in this paper. In this processing, we adhere to the theory that cone cells separate the channel information. The pixel-based scanning determines the information from the photoreceptors, as represented by the color scale of the pixel. A single receptive field, processed by AVS as shown in Figure 2a, is now divided into channels, as illustrated in Figure 3a.

There are four layers for a dendritic neuron model, which process the signals from inputs.

The synaptic layer receives input signals from bipolar cells and horizontal cells, which have processed the visual stimuli into binary values (0 and 1), the proposed input format for the dendritic neuron model. It processes raw image data and feeds them into the subsequent layers. These inputs are processed by the activation function of the synapses, which have their own weights and biases. In this paper, we define the activation function as a sigmoid function. For a dendritic model that has *I* synapses and *J* branches and *M* outputs, the process on the *i*th synapse and *j*th branch for a specific output *m*, which is located at (a,b) pixel in the *c*th channel, can be described by the following formula:

(3) $$S_{ijm}^{{\left(a,b\right)}^{c}}\left(x_{i}\right)=\frac{1}{1+exp(-\frac{1}{d_{ijm}}\left(w_{ijm}^{c}x_{i}-q_{ijm}^{c}\right))}.$$

where $d_{ijm}$ is the distance parameter, which refers to the relative distance between the input and output signals and can be regarded as a hyperparameter defined by researchers. In our model, the learning dendritic neuron model, the ganglion cells’ inputs are integrated by the bipolar cells and horizontal cells after the vertical and horizontal On–Off response mechanism is employed.As a result, the inputs in this layer are defined as

(4) $$x_{i}=\left\{\begin{array}{c} H_{(a,b)i}^{c},\left(i\neq 5\right)\\ B_{(a,b)i}^{c},\left(i=5\right) \end{array}\right.,$$

Since the local receptive field is size 3×3, the number of the inputs should be 9.

The branch layer processes the output of the synaptic layer. Each branch applies nonlinear operations, resembling how dendrites in biological neurons perform localized computations. This layer is crucial for detecting spatial features by processing the inputs as follows:

(5) $$b_{jm}^{{\left(a,b\right)}^{c}}=\prod_{i=1}^{I}S_{ijm}^{{\left(a,b\right)}^{c}}.$$

In the membrane layer, the outputs from the dendritic branches are integrated, simulating the summation of signals across the neuron’s membrane. This layer operates by

(6) $$u_{m}^{{\left(a,b\right)}^{c}}=\sum_{j=1}^{J}v_{jm}^{c}b_{jm}^{{\left(a,b\right)}^{c}}.$$

where $v_{jm}^{c}$ is the weight of the corresponding branch in this channel.

Finally, the soma integrates all the signals from the membrane layer and generates the neuron’s output. This step represents the point at which the neuron decides whether or not to fire based on a comparison between the accumulated input and the soma’s threshold, $\theta_{m}^{c}$. The activation function for the soma is also a sigmoid function, expressed as

(7) $$O_{m}^{{\left(a,b\right)}^{c}}=\frac{1}{1+exp(-\lambda_{m}^{c}\left(u_{m}^{{\left(a,b\right)}^{c}}-\theta_{m}^{c}\right))}.$$

where $\lambda_{m}^{c}$ is the slope parameter of the soma.

After going through the processing stages in the dendritic neuron model applied to ganglion cells, the activation status of the *m*th output for the local receptive field can be determined. The synaptic layer initially processes the input signals from bipolar and horizontal cells, transforming them through weighted synaptic activation. These transformed signals are then processed by the branch layer, which applies nonlinear operations, followed by the membrane layer that integrates the outputs from the branches. Finally, the soma layer performs the final integration and determines whether the neuron fires by comparing the accumulated signals to its threshold. Through these steps, the model effectively computes the activation for detecting visual features in the local receptive field at the *m*th output. The sequence of processes occurring in the ganglion cells for each individual receptive field is detailed in Figure 3b, which likely illustrates the neural activity and signal transmission within the ganglion cells corresponding to the receptive field. The connection positions are influenced by the synaptic weight and bias, as shown in Figure 3c.

### 2.5. Multi-Channel Processing Based on Dendrite Neuron Model to LGN

After determining the responses for each channel, the results are combined to form a final integrated response that reflects the color-specific motion or object boundary information. Each pixel in the image is evaluated based on its motion status in each channel, allowing for a more comprehensive understanding of motion and color shifts. The lateral geniculate nucleus (LGN) processes multi-channel color information by receiving inputs from retinal ganglion cells sensitive to different color channels [31]. These inputs are segregated into parvocellular layers for chromatic processing, where contrast and spatial filtering refine the signals. The LGN then relays the processed color information to the visual cortex for further interpretation, maintaining the distinction between color channels throughout the process [32].

By extending the On–Off response mechanism to multiple channels, the model can effectively detect changes in color and motion for each pixel in RGB images, enabling more accurate and robust visual perception. Since the lateral geniculate nucleus (LGN) integrates spatial information from all three color channels, we introduce the dendritic neuron model to perform this integration. Specifically, the LGN requires the activation of all three channels (RGB) for the final state to be fully activated, and the dendritic model can simulate this phenomenon effectively. In our model, because all three channels must be activated for the LGN’s spatial integration, the synaptic connections do not require learning, as they are predefined to always contribute. This reflects the biological necessity for all channels to be involved in the integration process.

**Figure 4:**
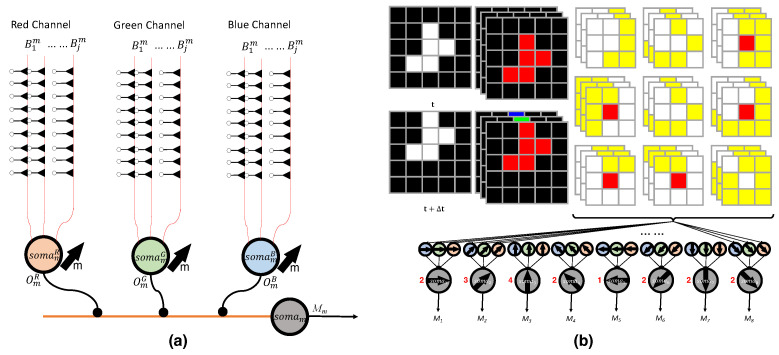
(**a**) Diagram illustrating the activation in each channel, corresponding to a single direction for the certain local receptive field. (Note: The figure omits the explicit index of the receptive field’s position for clarity.) (**b**) Example showing the processing of a 5×5 image before and after motion, showing how RGB channels separate the information and activate vertical and horizontal On–Off responses and how the LGN integrates these responses to determine the overall motion direction.

As shown in our previous work, linear summation of information does not require redundant branches, so we simplify the branch layer to a single branch [18]. The membrane and soma layers then perform the final integration of the information across channels, and this can be defined as follows:

(8) $$M_{m}^{\left(a,b\right)}=\frac{1}{1+exp(-\lambda_{m}\left(\prod_{c=1}^{3}O_{m}^{{\left(a,b\right)}^{c}}-\theta_{m}\right))}.$$

where $M_{m}^{\left(a,b\right)}$ represents the activation state in direction *m* for the local receptive field centered at pixel (a,b). Figure 4a, illustrates how the activation in each channel corresponding to direction $O_{m}^{(a,b)c}$ ultimately determines the overall direction $M_{m}^{\left(a,b\right)}$ for the local receptive field at (a,b).

This equation ensures that only when all three channels contribute sufficiently is the final integrated response triggered, allowing the LGN to detect complex color-specific motion and spatial boundaries accurately.

After the processing of LGN, the corresponding neurons located at (a,b) give the activation for all the *M* motion directions. Then the visual cortex analyzes the outputs of LGN and integrates the final output. Figure 4b illustrates how the proposed model is applied to an A×B-pixel image, analyzing each receptive field independently to determine the overall motion direction. The detection result of each direction can be regarded as the following equation:

(9) $$D_{m}=\sum_{a=1}^{A}\sum_{b=1}^{B}M_{m}^{\left(a,b\right)}.$$

By systematically applying the dendritic neuron model to each pixel’s local receptive field and combining the responses from all three color channels (RGB), the global model integrates spatial and color-specific information to detect the predominant direction of motion and object boundaries. The model replicates the processing mechanism of the lateral geniculate nucleus (LGN), where activation requires input from all channels. Each local computation contributes to the global interpretation of motion and color shifts across the entire image. By aggregating these local responses, the model forms a cohesive understanding of the overall motion and visual features, effectively combining chromatic and spatial information into a unified motion detection framework.

To illustrate the operation of the LGN-based three-channel motion direction detection AVS, we present an example. As shown in Figure 4b, we use a 5×5 image consisting of frames before and after motion. For simplicity, we consider extreme cases with only black (all channels at 0) and white (all channels at 255). The image contains a white object of size 5 against a black background. Due to the role of cone cells, each channel is processed separately into RGB channels. Since the values are either 0 or 255, the activation in each channel’s direction is identical. In the activation map, white indicates no activation, red represents the activated vertical On–Off response, and yellow indicates the activated horizontal On–Off response. These signals are first received by the $soma_{m}^{c}$ of the respective channel, and then the activations are integrated by the $soma_{m}$ of the LGN, which consolidates the activations across all three channels. It can be observed that the local activation signal on $M_{3}^{\left(a,b\right)}$ is the strongest, so the system concludes that the overall direction $D_{3}$ is activated.

### 2.6. Learning Algorithm and System Diagram

Our motion direction detecting dendritic neuron is designed to learn and adapt based on input stimuli. Blakemore suggests that motion direction selectivity develops due to external factors from birth, a notion supported by contemporary research and used in previous dendritic neuron model-based artificial visual systems [4,6,18]. The synaptic connection states of the dendrites in our model, following this theory, are determined through learning. We use the cross-entropy loss function to train the model for motion direction detection, defined as

(10) $$E=-\sum_{m=1}^{8}T_{m}\log\left(\bar{D_{m}}\right)$$

where *E* is the cross-entropy loss; $\bar{D_{m}}$ is the normalized overall output for motion direction detection in a group of A×B input images; and $T_{m}$ denotes the teacher’s signal for the correct motion direction. This loss function helps optimize the model by minimizing the difference between the predicted and true motion directions.

Following Blakemore’s theory, we restructure the synaptic layer to better align with the dendritic neuron model’s learning process. We adjust the synaptic connection parameters $w_{ijm}^{c}$ and $q_{ijm}^{c}$ to minimize the error in motion direction detection. The updates to these parameters are computed using gradient descent, where the change in the weights is

(11) $$\Delta w_{ijm}^{c}=-\eta \frac{\partial E}{\partial w_{ijm}^{c}},$$

and the change in biases is

(12) $$\Delta q_{ijm}^{c}=-\eta \frac{\partial E}{\partial q_{ijm}^{c}},$$

with η as the learning rate. By applying the chain rule, we derive the following gradient expressions:

(13) $$\frac{\partial E}{\partial w_{ijm}^{c}}=\frac{\partial E}{\partial \bar{D_{m}}}\cdot \frac{\partial \bar{D_{m}}}{\partial D_{m}}\cdot \frac{\partial D_{m}}{\partial M_{m}^{\left(a,b\right)}}\cdot \frac{\partial M_{m}^{\left(a,b\right)}}{\partial O_{m}^{{\left(a,b\right)}^{c}}}\cdot \frac{\partial O_{m}^{{\left(a,b\right)}^{c}}}{\partial b_{jm}^{{\left(a,b\right)}^{c}}}\cdot \frac{\partial b_{jm}^{{\left(a,b\right)}^{c}}}{\partial S_{ijm}^{{\left(a,b\right)}^{c}}}\cdot \frac{\partial S_{ijm}^{{\left(a,b\right)}^{c}}}{\partial w_{ijm}^{c}},$$

and similarly for the bias:

(14) $$\frac{\partial E}{\partial q_{ijm}^{c}}=\frac{\partial E}{\partial \bar{D_{m}}}\cdot \frac{\partial \bar{D_{m}}}{\partial D_{m}}\cdot \frac{\partial D_{m}}{\partial M_{m}^{\left(a,b\right)}}\cdot \frac{\partial M_{m}^{\left(a,b\right)}}{\partial O_{m}^{{\left(a,b\right)}^{c}}}\cdot \frac{\partial O_{m}^{{\left(a,b\right)}^{c}}}{\partial b_{jm}^{{\left(a,b\right)}^{c}}}\cdot \frac{\partial b_{jm}^{{\left(a,b\right)}^{c}}}{\partial S_{ijm}^{{\left(a,b\right)}^{c}}}\cdot \frac{\partial S_{ijm}^{{\left(a,b\right)}^{c}}}{\partial q_{ijm}^{c}},$$

According to the formulas, some parts can be simplified as

(15) $$\frac{\partial E}{\partial \bar{D_{m}}}=-\frac{T_{m}}{\bar{D_{m}}},$$

(16) $$\frac{\partial \bar{D_{m}}}{\partial D_{m}}\cdot \frac{\partial D_{m}}{\partial M_{m}^{\left(a,b\right)}}=AB\cdot \frac{1}{AB}=1,$$

(17) $$\frac{\partial M_{m}^{\left(a,b\right)}}{\partial O_{m}^{{\left(a,b\right)}^{c}}}=\frac{\lambda_{m}}{{\left(e^{\frac{\lambda_{m}\left(O_{m}^{{\left(a,b\right)}^{c}}-\theta_{m}\right)}{2}}+e^{-\frac{\lambda_{m}\left(O_{m}^{{\left(a,b\right)}^{c}}-\theta_{m}\right)}{2}}\right)}^{2}}=\frac{\lambda_{m}}{4\cosh^{2}\left(\frac{\lambda_{m}\left(O_{m}^{{\left(a,b\right)}^{c}}-\theta_{m}\right)}{2}\right)},$$

(18) $$\frac{\partial O_{m}^{{\left(a,b\right)}^{c}}}{\partial b_{jm}^{{\left(a,b\right)}^{c}}}=\frac{v_{jm}^{c}\lambda \exp\left(-\lambda_{m}^{c}\left(v_{jm}^{c}-\theta_{m}^{c}\right)\right)}{{\left(1+\exp\left(-\lambda_{m}^{c}\left(v_{jm}^{c}-\theta_{m}^{c}\right)\right)\right)}^{2}},$$

(19) $$\frac{\partial b_{jm}^{{\left(a,b\right)}^{c}}}{\partial S_{ijm}^{{\left(a,b\right)}^{c}}}=\prod_{\alpha \neq i}S_{\alpha jm}^{{\left(a,b\right)}^{c}},$$

(20) $$\frac{\partial S_{ijm}^{{\left(a,b\right)}^{c}}}{\partial w_{ijm}^{c}}=\frac{x_{i}\exp\left(-\frac{1}{d_{ijm}}\left(w_{ijm}^{c}x_{i}-q_{ijm}^{c}\right)\right)}{d_{ijm}{\left(1+\exp\left(-\frac{1}{d_{ijm}}\left(w_{ijm}^{c}x_{i}-q_{ijm}^{c}\right)\right)\right)}^{2}},$$

(21) $$\frac{\partial S_{ijm}^{{\left(a,b\right)}^{c}}}{\partial q_{ijm}^{c}}=-\frac{\exp\left(-\frac{1}{d_{ijm}}\left(w_{ijm}^{c}x_{i}-q_{ijm}^{c}\right)\right)}{d_{ijm}{\left(1+\exp\left(-\frac{1}{d_{ijm}}\left(w_{ijm}^{c}x_{i}-q_{ijm}^{c}\right)\right)\right)}^{2}}.$$

According to research, the intrinsic properties of retinal cells and their genetic programming play a key role in shaping these mechanisms [11]. The segregation of On and Off pathways initiates at the photoreceptor layer and continues through bipolar and ganglion cells. These genetically guided processes are fundamental for early visual functionality, ensuring that basic visual processing is in place from birth [9]. In this structure, the dendritic model specifically captures the learning process at the synaptic level, where synaptic connections mimic biological behavior by integrating inputs from multiple branches. Since dendrites focus on localized information processing, the adjustments to the synaptic parameters directly affect how inputs are combined and transmitted across the branches and membranes. Each term in the chain rule represents the contribution of different layers—synapse, branch, membrane, and soma—to the overall error. The separated differential formula reinforcing the idea that the dendritic neuron model captures the complex integration of spatial and color information in motion detection.

**Figure 5:**
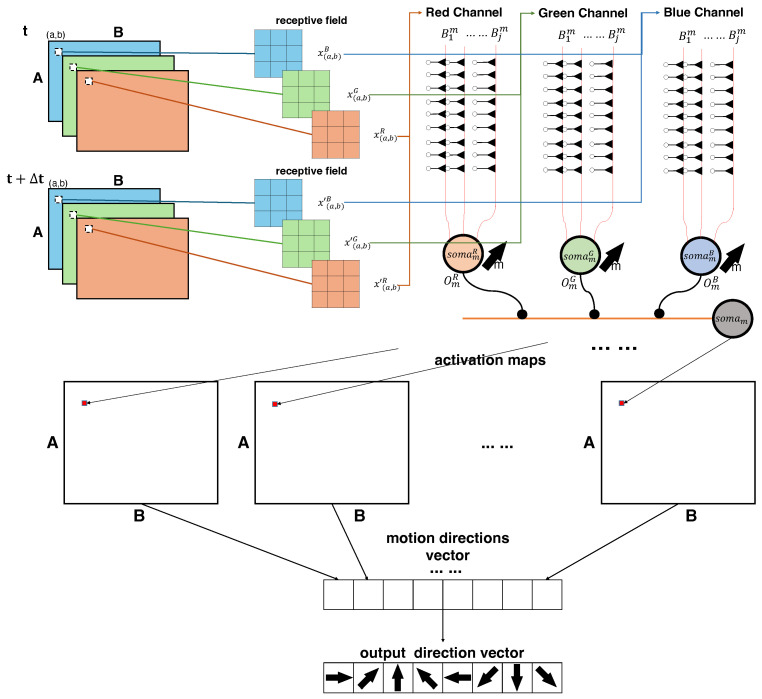
The whole diagram of the motion direction detection system.

We illustrate the entire process of motion direction detection in Figure 5, where our AVS closely mimics biological processing pathways. First, cone cells within the photoreceptor layer detect and encode information for each color channel separately. Next, bipolar cells and horizontal cells in the retina generate On–Off responses to detect horizontal and vertical motion changes between consecutive images. These signals are then processed by ganglion cells within the retina, which integrate motion direction information within each individual channel. The LGN in the primary cortex subsequently integrates multi-channel information, forming a motion direction judgment within the current local receptive field. These local motion judgments are then combined to form an overall assessment of motion direction, which is matched with the labels provided during training. In this study, predictions are generated through feedforward processing, while backpropagation is used to update model parameters based on prediction errors, enhancing the model’s motion direction detection capabilities. This learning process aligns with Blakemore’s theory on motion direction selectivity developed through external stimuli.

Each term in the chain rule represents the contribution of different layers—synapse, branch, membrane, and soma—to the overall error. These layers are parameterized to mimic biological processes, as summarized in Table 1. By outlining these relationships, we emphasize how the dendritic neuron model incorporates localized and global signal integration, mirroring neural mechanisms.

This table highlights the interplay between computational parameters and biological counterparts, underscoring the model’s biological plausibility. By explicitly linking synaptic weights, branch potentials, and membrane dynamics to their biological functions, we aim to bridge the gap between technical details and their conceptual inspirations. These parameters, when optimized through backpropagation, enhance AVS’s ability to detect motion direction effectively.

## 3. Simulation Results

### 3.1. Comparison Models

To address the challenges of processing multi-channel, multi-image data, we carefully selected three advanced CNN architectures: EfficientNet (EfN), ResNet, and ConvNeXt. These models were chosen based on their strong image processing performance. To address the balance between computational efficiency and accuracy, ensuring that they can handle the complexity of our dataset without introducing excessive overhead or overfitting, the choice among their various structures is also important.

EfN is specifically designed for efficiency in handling large batches of data. Its architecture, which uses compound scaling to balance network depth, width, and resolution, allows it to process more data per batch while reducing training time and memory usage. This helps minimize redundant loss during training, enabling the model to handle our dataset’s size efficiently.

By choosing a smaller variant, such as EfN-B0 in this paper, we maintain the ability to process more samples simultaneously without compromising on accuracy. EfN-B0 consists of 16 layers with depthwise separable convolutions, batch normalization, and squeeze-and-excitation blocks, which reduce computational complexity while maintaining accuracy. It also scales parameters based on image size, width, and depth, providing a lightweight and scalable solution [33]. It is also particularly relevant as a benchmark due to its lightweight design and its widespread adoption for resource-constrained tasks as the smallest variant of EfN. This focus on computational efficiency aligns with the design goals of AVS, which also prioritizes reducing resource requirements while maintaining high performance in complex datasets. In conclusion, we chose EfN-B0 for its optimized trade-off between computational efficiency and accuracy.

ResNet, with its residual connections, helps tackle the problem of deep networks struggling with vanishing gradients, particularly important in learning the complex patterns in our motion detection dataset. This architecture allows us to train deeper models without significantly increasing the risk of overfitting. This robustness to gradient degradation is complemented by AVS’s dendritic-inspired architecture, which provides efficient feature integration while maintaining adaptability to diverse visual conditions. Additionally, ResNet-50 strikes a balance between being powerful enough to extract meaningful features while being moderate in parameter size, which reduces overfitting risks that typically arise from using excessively complex models on relatively small datasets. As a result, ResNet-50 is employed in this paper as comparison model. ResNet-50 consists of 50 layers, including 48 convolutional layers and 2 fully connected layers, with shortcut connections that allow for easier gradient flow during backpropagation. The network is structured into multiple residual blocks, each containing two 3×3 convolutional layers followed by batch normalization and ReLU activation functions [34]. Furthermore, its inclusion allows us to compare our AVS against a well-established baseline model widely used in visual recognition and feature extraction.

ConvNeXt is a modern CNN that improves on traditional architectures, offering a balance of performance and lightweight design. We chose the ConvNeXt-Tiny variant to ensure that the model complexity is kept under control, avoiding overfitting. The simplicity of this architecture also reduces the chances of overcomplication in model training, enabling faster convergence. Its ability to process spatial and temporal features in a streamlined way supports our goal of handling large batches while avoiding unnecessary computational overhead. ConvNeXt-Tiny consists of 28 layers, built on a hierarchical architecture with a large receptive field, depthwise convolutions, and layer normalization, improving efficiency and effectiveness [35]. This version of ConvNeXt-Tiny maintains the simplicity of a traditional CNN while incorporating insights from vision transformers, leading to improved feature extraction capabilities without adding excessive complexity. The architectural simplicity and lightweight design of ConvNeXt-Tiny make it a relevant benchmark to evaluate the resource efficiency of AVS in multi-channel motion detection tasks. ConvNeXt-Tiny’s incorporation ensures a comprehensive evaluation across both traditional and contemporary architectures.

By selecting these models, we achieve a balanced comparison across different CNN generations, each offering distinct design philosophies and trade-offs in efficiency, complexity, and feature extraction capabilities. This diverse benchmarking strategy allows us to highlight AVS’s advantages in computational efficiency and adaptability to multi-channel motion detection, especially under constrained training data conditions.

Given the multi-channel nature of our dataset, where two consecutive frames of 32×32×3 images are processed, a common approach is to concatenate the images along the channel dimension and feed the combined data into the network. This method ensures both spatial and temporal consistency, allowing the CNN to efficiently capture motion-related features while leveraging the capacity of these advanced architectures to extract meaningful patterns. The models we chose have the ability to concatenate two consecutive images along the channel dimension. This technique ensures that both spatial and temporal features are captured simultaneously within a unified framework. This allows the models to efficiently process the motion-related data without redundancy. By simplifying the network structure where possible, we further ensure that the models can handle a greater volume of data in each batch, reducing the computational load per batch and enhancing the overall training process. This also helps in preventing overfitting, as these models are tailored to operate within the resource constraints of our dataset and avoid introducing excessive model complexity.

In addition to the concatenation method, we also experimented with optical flow and difference images in preprocessing. Optical flow is based on the perception of relative motion in the retina, while difference images relate to how the visual system detects changes in light intensity over time. However, empirical results showed that datasets processed with optical flow and difference imaging struggled to achieve optimal accuracy when used with CNNs. These results suggest that these approaches emphasized only a narrow subset of motion characteristics, overlooking key features that capture both global and local structures. While object direction detection ideally minimizes dependency on shape or color, these attributes nevertheless contain important features that enhance motion recognition accuracy. Therefore, we ultimately adopted the image concatenation method to ensure the retention of all critical image features during processing.

While these CNN models effectively process concatenated image inputs to capture motion-related features, they often struggle with redundant data and variability in object–background combinations. Unlike CNNs, AVS leverages biologically inspired mechanisms that mimic the behavior of retinal ganglion cells and the LGN. This allows AVS to dynamically integrate spatial and temporal information, reducing redundancy while enhancing adaptability to diverse visual inputs. Such mechanisms enable AVS to outperform traditional CNNs in scenarios where high-contrast sensitivity and real-time adaptation are critical.

### 3.2. Dataset Statement

It is important to demonstrate the effectiveness and applicability of our proposed dendritic neuron model in detecting motion direction. Therefore, we designed a custom dataset that simulates object motion in various scenarios. The dataset consists of images where motion detection is critical, constructed with multi-channel (sized 32×32×3) square images that reflect object movement under different lighting and background conditions. Each channel corresponds to RGB encoding, with pixel values ranging from 0 to 255 in uint8 format.

For the dataset creation, we initialized each pixel with values representing distinct categories for both objects and backgrounds. Specifically, the images are classified into three primary object forms: random, constant, and dark. “Random” means that each pixel is assigned a value from 0 to 255 independently. “Constant” refers to pixels assigned a uniform random value between 1 and 255, while “Dark” keeps all pixel values at 0. This categorization results in the following eight object–background combinations:

(1) DL (dark object, light background);
(2) DR (dark object, random background);
(3) LD (light object, dark background);
(4) LL (light object, light background);
(5) LR (light object, random background);
(6) RD (random object, dark background);
(7) RL (random object, light background);
(8) RR (random object, random background).

Note that the case of both object and background being dark (DD) is omitted, as it holds no meaningful information.

**Figure 6:**
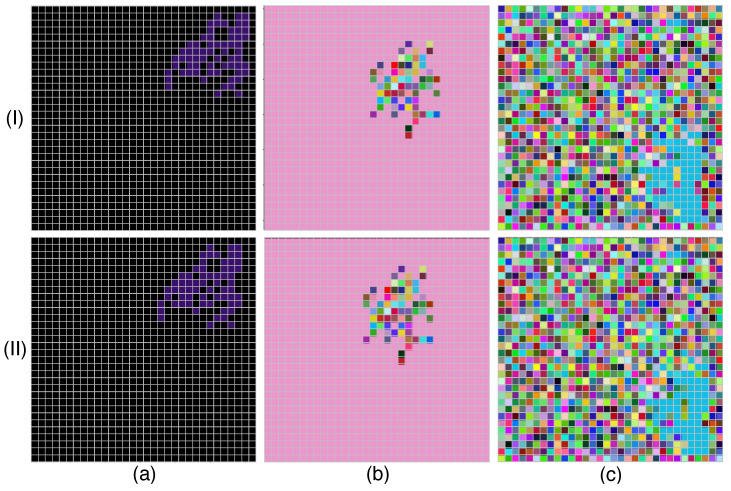
(**I**) Before motion at time *t*; (**II**) after motion at time t+Δt. (**a**–**c**) Images from different datasets.

Each object is randomly placed in the image, and its pixels are connected, forming a coherent structure. We varied object sizes across eight distinct categories: 1, 2, 4, 8, 16, 32, 64, and 128 pixels. To simulate motion, the object moves by one pixel in a random direction between successive frames, generating two 32×32×3 images. Each motion direction is represented by 500 image pairs for every object size, resulting in a total of 4000 groups per size, the image before motion at time *t* and after motion at time t+Δt. The samples of the dataset are shown in Figure 6.

This dataset allows our dendritic neuron model to fully leverage its multi-channel processing capabilities by simulating real-world motion scenarios across multiple color channels. The design of this dataset also reflects practical use cases, such as robotic navigation and surveillance. For example, high-contrast scenarios like DL (dark object on light background) mimic environments where objects are easily distinguishable, such as detecting a moving person in a brightly lit hallway. Conversely, configurations like DR (dark object on random background) simulate visually noisy environments, akin to detecting motion in cluttered scenes or low-light settings. This diversity in object–background contrasts helps evaluate a model’s adaptability to real-world challenges in motion detection, such as varying light conditions and complex visual patterns. This dataset provides a basis for evaluating how well different models, including our dendritic-neuron-based approach, can process multi-channel color information and detect motion under varied lighting and object–background conditions. Furthermore, the object–background combinations allow us to analyze how model performance varies depending on dataset characteristics. For instance, configurations with greater contrast (e.g., DL and LD) are expected to yield higher accuracies across models due to the prominence of object boundaries. In contrast, configurations like RR (random object on random background) introduce higher levels of visual complexity and noise, posing significant challenges for motion detection. CNN models, which rely on global convolutional kernels, are particularly susceptible to noise introduced by random backgrounds, as the convolution operation aggregates features across the entire image. This can cause the kernels to lose focus on relevant motion patterns amidst background randomness, leading to decreased accuracy. Conversely, the AVS model’s biologically inspired architecture, which combines local feature extraction with global integration, allows it to better isolate motion-related features while minimizing the impact of background noise. As a result, AVS demonstrates greater robustness in configurations like RR and LR, where visual complexity is high, and contrast is low. This distinction highlights the advantage of AVS’s localized processing approach in handling diverse and challenging scenarios. Subsequent sections explore the performance of this model in comparison with other approaches.

### 3.3. Cross Validation

In our cross-validation approach, we train each neural network model on one of the eight distinct dataset configurations, then evaluate its performance on the remaining seven configurations. This methodology allows us to systematically test each model’s generalization ability across various visual conditions, where each dataset configuration represents different combinations of object and background contrasts (such as light, dark, or random-pattern-based objects and backgrounds). For each training session, we select a single dataset and hold the other seven for testing, providing a thorough assessment of each model’s robustness across diverse settings.

This approach ensures that we capture how well a model trained in one specific environment performs when exposed to new, unseen configurations. Such a setup is particularly relevant for our application, as it mimics real-world scenarios where visual input can vary widely in terms of object and background characteristics. Additionally, our method reveals the models’ adaptability and identifies which architectures are more effective at handling variations in visual scenes, supporting the biological realism of our dendritic-neuron-based motion direction detection model by simulating how biological systems process varied inputs. The result is shown in Table 2.

In this experiment, the cross-validation results for four different models (AVS, EfN-B0, ResNet-50, and ConvNeXt-tiny) are presented across eight dataset configurations. Each configuration represents a different contrast between objects and backgrounds, such as dark or random-patterned backgrounds.

EfN-B0 performs poorly across testing configurations, often achieving less than 20% accuracy, particularly in testing configurations distinct from the training one. While it reaches close to 100% training accuracy in some configurations, its poor performance in cross-configuration testing suggests limited adaptability to new backgrounds and object combinations. ResNet-50 and ConvNeXt-tiny also achieve low cross-configuration accuracy, mostly below 20% and in some cases close to chance-level accuracy, indicating weak adaptability to diverse visual conditions.

On the other hand, the AVS model consistently demonstrates nearly 100% training accuracy across all configurations, suggesting strong stability and effectiveness in identifying visual features in the training data. Importantly, it also achieves high accuracy across various testing configurations, particularly in configurations with different backgrounds. For instance, when trained on configuration DL, AVS achieves high testing accuracy on configurations LL and RL (99.16% and 99.70%), highlighting its adaptability to different visual backgrounds. Although the accuracy may drop in more contrasting configurations, AVS typically maintains over 50% accuracy, outperforming chance-level classification and suggesting substantial generalization capability.

In summary, in the cross-validation experiments, AVS demonstrates exceptional cross-configuration adaptability unmatched by traditional convolutional models. Its ability to maintain high accuracy in diverse visual environments points to greater robustness and biological plausibility, mirroring how real-world biological systems manage diverse inputs.

The AVS model leverages its dendritic-neuron-based architecture, which combines localized feature extraction and global integration, allowing it to generalize effectively across diverse datasets. In contrast, CNN models like ResNet-50 and ConvNeXt struggle in high-noise scenarios due to their reliance on global convolutions, which can amplify background randomness and lead to reduced accuracy. Basically, statistical tests, including paired t-tests between AVS and CNN models across dataset configurations, confirm significant performance differences (p≤0.001≤0.05), particularly in challenging configurations like RR and LR. These results highlight AVS’s robustness in real-world-like noisy environments. Furthermore, the adaptability of AVS to various input configurations without requiring architectural changes demonstrates its practical scalability for diverse applications.

### 3.4. Different Training Data Ratio

Demonstrating the robustness of a model and its capacity for effective learning even when the training dataset is significantly limited is important for testing a model’s performance. As a result, we designed an experiment with various training-to-testing data ratios. This experiment evaluates the model’s adaptability across four dataset divisions: 75:25, 50:50, 10:90, and 5:95. By comparing AVS with other commonly used vision processing models, AVS’s effectiveness in scenarios with limited training data, which are full of challenges for most models, can be highlighted. This analysis is crucial in establishing the model’s potential applicability in real-world cases where labeled data are often scarce or difficult to obtain. The result is shown in Table 3.

In the results shown above, AVS consistently achieves high accuracy across all train–test splits, demonstrating a superior capacity to generalize from limited data. With a 75:25 split, AVS achieves both a high training accuracy and test accuracy of nearly 100%. Similarly, with a 50:50 split, AVS maintains high accuracy for both training and test. Even with a significantly reduced training dataset, such as in the 10:90 and 5:95 splits, AVS achieves test accuracies of over 95%, respectively. These results demonstrate that AVS remains reliable and capable of learning effectively even when trained on a minimal amount of data.

In contrast, other models like EfN-B0, ResNet50, and ConvNeXt exhibit a significant drop in test accuracy as the training data decrease. For example, EfN-B0 shows a test accuracy of about 60% at the 75:25 split, but this quickly declines to lower than 15% in the 10:90 and 5:95 splits. Similarly, ResNet50 and ConvNeXt show very limited generalization when training data are reduced, with test accuracies around or below 15% for most splits, close to a chance-level accuracy.

For further comparison, we conducted additional experiments to analyze the computational efficiency of the AVS and CNN-based models. These tests involved running the models with identical image sizes, GPUs, devices, and batch sizes to ensure fairness. Using the 75:25 training-to-testing data ratio, where all models demonstrated learning capability, we evaluated the average training duration and memory usage over ten runs. The results are summarized in Table 4.

These results indicate that AVS requires substantially fewer parameters and memory while maintaining competitive training times. For instance, AVS uses only 432 parameters compared to ConvNeXt’s 27.8 million. Its lightweight architecture ensures faster training times (approximately less than 1 s per epoch) and minimal memory usage. This efficiency positions AVS as a practical alternative for applications with resource constraints, such as edge devices or real-time processing. Similar trends are observed in the cross-validation experiments, where AVS consistently demonstrated superior performance with minimal computational overhead. In comparison, while CNN models like ResNet50 and ConvNeXt demonstrate strong performance in large-scale data processing tasks, their significant memory and computational overhead limit their applicability in low-resource scenarios. This analysis underscores AVS’s robustness and adaptability, even under constrained resources, making it particularly suitable for real-world applications with limited hardware capabilities.

Considering about the experimental results, AVS performed better than other models. The superior performance of AVS across various datasets can be attributed to its dendritic-inspired architecture, which closely mimics biological visual systems. This model’s localized processing capability allows it to effectively extract and integrate spatially relevant features, particularly in datasets with significant variability in background patterns or high object–background contrast. Unlike CNNs, which rely on global convolutions that may amplify noise in random patterns, AVS processes information within adaptive receptive fields, enabling better generalization. Moreover, AVS’s focus on edge and motion contrast aligns with the dataset’s nature, where object–background differentiation plays a critical role. These design advantages ensure AVS maintains high performance across configurations where CNN models face challenges due to their reliance on fixed kernel sizes and global feature extraction.

Overall, this experiment highlights AVS’s robustness and adaptability, even with minimal training data and limited maximum memory storage, positioning it as a highly effective model for scenarios with limited labeled data.

## 4. Conclusions

This study introduced a dendritic-neuron-based AVS that emulates biological visual pathways to detect motion direction in RGB images. By modeling the functions of photoreceptor, bipolar, horizontal, and ganglion cells, our AVS processes spatial–temporal information across multiple color channels. This biomimetic approach enables effective motion detection with a reduced requirement for extensive data, making it both resource-efficient and accurate. Our findings validate the AVS model’s alignment with Blakemore’s theory on motion selectivity, establishing a meaningful connection between biological vision mechanisms and artificial systems.

While AVS achieves robustness and accuracy, certain limitations present opportunities for future enhancement. The current design primarily addresses simplified visual scenes; thus, its performance in more complex and dynamic environments, such as scenes with varying lighting or occlusions, requires further investigation. Expanding the model’s adaptability to handle such conditions could enhance its practical applications in real-world scenarios.

For future research, several directions could enhance both AVS’s functionality and applicability. Introducing additional computational strategies may further improve the model’s real-time processing capabilities. Additionally, extending the model to incorporate other visual features, such as depth perception or object segmentation, would create a more comprehensive visual processing system. Finally, enabling AVS to adapt to environmental changes through mechanisms such as reinforcement learning could increase its robustness and flexibility across diverse visual scenes.

In summary, this AVS model provides a promising foundation for biologically inspired, multi-channel visual processing models that efficiently bridge artificial intelligence and biological vision systems. With continued advancement, this model holds potential applications in fields such as robotics, autonomous systems, and medical image analysis, where reliable, data-efficient motion detection is essential.

## Figures and Tables

**Table 1:** Biological explanation of the parameters in AVS.

Parameter	Explanation
$x_{\left(a,b\right)}^{c}$	Light intensity detected by photoreceptor cells.
$\epsilon_{V}$	Determines if the center pixel corresponds to motion.
$\epsilon_{H}$	Assesses if the center pixel and nearby pixels belong to the same moving object.
$w_{ijm}^{c}$	Synaptic weight applied to bipolar cell inputs.
$q_{ijm}^{c}$	Synaptic bias determining bipolar cell input recognition.
$d_{ijm}$	Distance between the synapse and bipolar cell.
$S_{ijm}^{{\left(a,b\right)}^{c}}\left(x_{i}\right)$	Synapse output signal, input to the branch layer.
$b_{jm}^{{\left(a,b\right)}^{c}}$	Branch layer output, input to the membrane layer.
$u_{m}^{{\left(a,b\right)}^{c}}$	Membrane layer output, input to the soma layer.
$v_{jm}^{c}$	Branch signal strength.
$O_{m}^{{\left(a,b\right)}^{c}}$	Soma layer output, input to LGN.
$M_{m}^{\left(a,b\right)}$	LGN output indicating motion direction.
$D_{m}$ & $\bar{D_{m}}$	Final motion direction judgment by the brain.

**Table 2:** Cross validation of different dataset for proposing models. The yellow pixels indicate identical learning outcomes achieved by the model when trained and tested on the same dataset configuration.

Model	Train Data	Train Acc	Test Acc							
			**DL**	**DR**	**LD**	**LL**	**LR**	**RD**	**RL**	**RR**
AVS	**DL**	98.76±0.19%	98.65±0.19%	45.00±16.6%	79.08±2.66%	99.16±0.33%	44.84±17.2%	82.72±2.52%	99.70±0.15%	58.67±12.4%
	**DR**	99.48±0.10%	64.38±0.75%	99.42±0.12%	100.0±0.00%	65.30±1.17%	99.40±0.17%	100.0±0.00%	68.64±1.33%	99.60±0.08%
	**LD**	99.38±0.29%	52.69±13.7%	96.96±2.95%	99.41±0.36%	53.18±13.6%	96.39±2.88%	99.81±0.12%	63.59±12.0%	97.84±2.31%
	**LL**	99.64±0.19%	98.43±0.44%	90.75±7.13%	77.74±3.23%	99.54±0.24%	90.53±7.12%	83.20±3.16%	99.78±0.13%	91.65±6.34%
	**LR**	99.47±0.07%	64.24±0.92%	99.48±0.15%	100.0±0.00%	64.93±0.90%	99.48±0.17%	100.0±0.00%	68.20±1.12%	99.58±0.08%
	**RD**	99.71±0.05%	46.85±10.2%	98.05±0.45%	96.41±0.99%	45.82±9.06%	97.91±0.56%	99.66±0.14%	58.66±8.15%	99.32±0.15%
	**RL**	99.62±0.04%	94.23±2.07%	88.08±5.42%	77.61±6.19%	94.03±2.00%	87.33±5.39%	90.52±3.31%	99.59±0.14%	92.60±4.31%
	**RR**	99.61±0.09%	63.24±3.05%	99.55±0.13%	95.85±4.75%	63.55±3.00%	99.49±0.11%	96.63±3.93%	67.52±2.42%	99.57±0.15%
EfN-B0	**DL**	99.68±0.05%	99.53±0.12%	15.00±1.41%	73.12±16.6%	85.48±1.77%	12.70±1.13%	73.93±15.6%	91.95±1.55%	12.74±0.58%
	**DR**	100.0±0.00%	12.88±1.04%	24.92±0.33%	12.65±0.81%	12.18±0.60%	13.33±0.65%	12.45±0.72%	12.60±1.07%	12.33±0.57%
	**LD**	100.0±0.00%	12.40±1.39%	12.65±0.70%	99.95±0.05%	12.29±0.89%	13.29±1.02%	99.91±0.06%	12.62±0.62%	12.18±0.56%
	**LL**	100.0±0.00%	99.02±0.25%	12.56±1.28%	99.70±0.17%	99.56±0.14%	11.89±0.72%	99.70±0.18%	99.74±0.11%	12.33±0.96%
	**LR**	100.0±0.00%	12.30±0.89%	12.82±0.93%	12.52±0.50%	12.55±1.04%	12.68±0.87%	12.43±1.08%	12.46±0.68%	12.55±0.57%
	**RD**	100.0±0.00%	12.76±1.05%	12.41±0.56%	99.92±0.04%	12.93±1.08%	13.01±0.82%	99.95±0.06%	12.82±0.87%	12.32±0.51%
	**RL**	100.0±0.00%	99.08±0.36%	12.66±0.97%	99.66±0.15%	98.61±0.28%	12.22±0.93%	99.81±0.01%	99.72±0.14%	12.61±0.86%
	**RR**	100.0±0.00%	12.58±0.96%	12.44±0.56%	12.64±0.66%	12.62±0.79%	12.76±0.69%	12.58±1.30%	12.57±0.65%	12.57±0.72%
ResNet-50	**DL**	99.66±0.06%	37.41±6.98%	17.86±1.67%	15.70±2.83%	18.21±1.97%	12.70±0.91%	15.78±3.21%	20.09±2.60%	12.64±0.68%
	**DR**	100.0±0.00%	14.12±1.25%	17.03±2.45%	12.20±0.86%	12.64±0.96%	12.71±0.45%	12.54±0.70%	12.34±0.63%	12.73±0.81%
	**LD**	99.99±0.02%	13.00±0.93%	12.05±1.00%	99.48±1.06%	12.50±1.07%	12.30±0.99%	99.48±0.93%	12.68±0.67%	12.51±0.76%
	**LL**	99.98±0.03%	15.31±1.22%	12.78±0.83%	14.92±1.30%	14.61±1.20%	12.73±0.65%	14.62±1.26%	15.20±1.52%	12.68±0.67%
	**LR**	100.0±0.00%	12.87±0.81%	12.69±0.47%	12.21±0.44%	12.72±0.59%	12.26±0.51%	12.54±0.53%	12.74±0.70%	12.32±0.68%
	**RD**	100.0±0.00%	12.99±1.14%	12.36±1.15%	99.36±0.40%	13.21±1.02%	12.46±0.77%	99.86±0.12%	12.74±0.94%	12.52±1.00%
	**RL**	99.99±0.02%	21.64±7.32%	12.55±0.91%	19.10±5.25%	20.08±5.91%	12.22±0.77%	19.24±5.62%	21.18±6.57%	12.47±0.56%
	**RR**	100.0±0.00%	12.68±0.74%	12.32±0.52%	12.67±0.83%	12.27±0.46%	12.50±0.57%	12.81±0.78%	12.16±0.91%	12.39±0.45%
ConvNeXt-tiny	**DL**	99.97±0.06%	99.55±0.13%	30.15±16.9%	13.04±1.64%	49.45±11.3%	13.93±1.33%	13.53±2.34%	67.12±11.2%	13.42±1.38%
	**DR**	99.98±0.03%	17.64±4.92%	16.81±4.35%	12.16±1.17%	13.49±1.70%	12.76±0.88%	11.75±0.99%	13.20±1.29%	12.60±0.57%
	**LD**	99.98±0.03%	12.35±1.01%	12.93±1.21%	99.92±0.02%	12.23±0.51%	12.58±0.48%	99.92±0.06%	12.49±0.59%	12.42±0.84%
	**LL**	99.09±0.65%	25.58±15.4%	12.52±0.80%	17.98±5.38%	27.60±18.7%	12.82±0.69%	18.50±5.88%	28.64±20.2%	12.99±0.92%
	**LR**	99.96±0.07%	12.31±0.45%	12.35±0.74%	12.81±0.78%	12.50±0.50%	12.30±0.90%	12.69±0.73%	12.44±0.57%	12.61±0.44%
	**RD**	100.0±0.00%	13.18±1.43%	13.16±1.39%	100.0±0.00%	12.77±0.85%	12.58±0.72%	99.96±0.04%	12.20±0.74%	12.56±0.88%
	**RL**	99.82±0.20%	69.71±9.24%	13.92±1.02%	28.85±9.79%	91.62±9.28%	13.84±1.19%	30.54±11.3%	94.77±7.01%	13.85±0.98%
	**RR**	99.74±0.53%	12.31±0.56%	12.84±0.58%	12.35±0.43%	12.46±0.74%	12.50±0.37%	12.28±0.65%	12.14±0.73%	12.86±0.43%

**Table 3:** Comparison of AVS with other vision processing systems on different training data and testing data ratios.

Model	Train Acc	Test Acc	Train Acc	Test Acc
Train–Test	75:25		50:50	
AVS	99.30±0.15%	99.19±0.16%	99.17±0.27%	99.03±0.32%
EfN-B0	99.97±0.23%	61.67±1.38%	99.93±0.06%	13.45±0.35%
ResNet50	97.40±2.50%	19.26±2.40%	99.96±0.00%	14.45±0.00%
ConvNeXt	99.80±0.23%	21.53±7.66%	99.86±0.07%	14.66±2.32%
Train–Test	10:90		5:95	
AVS	98.06±1.13%	96.77±1.69%	99.11±0.30%	96.14±0.78%
EfN-B0	100.0±0.00%	14.98±0.65%	100.0±0.00%	13.70±0.33%
ResNet50	12.03±1.11%	12.79±0.29%	12.66±1.29%	12.55±0.29%
ConvNeXt	99.93±0.08%	13.51±0.33%	13.80±1.65%	12.42±0.26%

**Table 4:** Comparison of AVS with CNN-based models in terms of computational efficiency. The yellow pixels indicate identical learning outcomes achieved by the model when trained and tested on the same dataset configuration.

Model	Parameters	Layers	Memory Usage	Average Duration
AVS	432	5	1 h 11 m 14 s	16.71%
EfN-B0	4017796	237	1 h 33 m 48 s	60.12%
ResNet50	23524424	50	13 m 36 s	68.53%
ConvNeXt	27822824	24	7 h 38 m 46 s	59.07%

## Data Availability

The data used in this study are of limited access but available for reasonable requirements. Interested parties may request access by contacting the corresponding author at yktodo@se.kanazawa-u.ac.jp. Access is subject to approval and compliance with confidentiality and ethical guidelines. The authors are committed to facilitating access within legal and ethical boundaries. Training Result—https://wandb.ai/ruriiiii/RGB_Motion_Simulation?nw=nwuserruriiiii (accessed on 15 November 2024) and Validation Result—https://wandb.ai/ruriiiii/RGB_Motion_Validation?nw=nwuserruriiiii (accessed on 15 November 2024) are available for reasonable requests. Dendritic neuron model-based AVS programs are available.
